# Feasibility of familial PSA screening: psychosocial issues and screening adherence

**DOI:** 10.1038/sj.bjc.6602959

**Published:** 2006-01-24

**Authors:** J Sweetman, M Watson, A Norman, Z Bunstead, P Hopwood, J Melia, S Moss, R Eeles, D Dearnaley, C Moynihan

**Affiliations:** 1 Academic Department of Radiotherapy, Institute of Cancer Research and Royal Marsden NHS Trust, Downs Road, Sutton, Surrey SM2 5PT, UK; 2 Department of Psychological Medicine, The Royal Marsden NHS Foundation Trust, Downs Road, Sutton, Surrey SM2 5PT, UK; 3 Department of Computing and Information, The Royal Marsden NHS Trust, and Institute of Cancer Research, Downs Road, Sutton, Surrey SM2 5PT, UK; 4 Department of Psycho-Oncology, The Christie Hospital, Manchester M20 4XB, UK; 5 Cancer Screening Evaluation Unit, Institute of Cancer Research Brookes Lawley Building, 15 Cotswold Road, Sutton, Surrey SM2 5NG, UK; 6 Translational Cancer Genetics Team, Institute of Cancer Research & Cancer Genetics Unit, Royal Marsden NHS Foundation Trust, Downs Road, Sutton, Surrey SM2 5PT, UK

**Keywords:** prostate cancer, psychological morbidity, screening, adherence, cancer distress

## Abstract

This study examined factors that predict psychological morbidity and screening adherence in first-degree relatives (FDRs) taking part in a familial PSA screening study. Prostate cancer patients (index cases – ICs) who gave consent for their FDRs to be contacted for a familial PSA screening study to contact their FDRs were also asked permission to invite these FDRs into a linked psychosocial study. Participants were assessed on measures of psychological morbidity (including the General Health Questionnaire; Cancer Worry Scale; Health Anxiety Questionnaire; Impact of Events Scale); and perceived benefits and barriers, knowledge; perceived risk/susceptibility; family history; and socio-demographics. Of 255 ICs, 155 (61%) consented to their FDRs being contacted. Of 207 FDRs approached, 128 (62%) consented and completed questionnaires. Multivariate logistic regression revealed that health anxiety, perceived risk and subjective stress predicted higher cancer worry (*P*=0.05). Measures of psychological morbidity did not predict screening adherence. Only past screening behaviour reliably predicted adherence to familial screening (*P*=0.05). First-degree relatives entering the linked familial PSA screening programme do not, in general, have high levels of psychological morbidity. However, a small number of men exhibited psychological distress.

Despite the mortality and rising incidence from prostate cancers, screening of the general male population remains controversial and there is insufficient evidence to recommend screening of the general male population given the lack of data demonstrating a mortality reduction (Frankel, 1997; Donovan *et al*, 2001). Randomised control trials are underway in Europe and the US. However, conclusive mortality analyses are not expected until approximately 2010 (Roobol and Schroder, 2003). In the UK, the National Health Service does not recommend routine population PSA screening. However, PSA testing is available for those men who request it and have been given detailed information in order to make an informed choice (Department of Health, 2000).

Regardless of uncertainties associated with general population PSA screening, targeted PSA screening of men at high-risk due to family history of prostate cancer may be of benefit (Catalona *et al*, 2002; Valeri *et al*, 2002). Familial risk could account for up to 10% of cases, the proportion being much higher at young ages: 43% in men aged 55 or less (Carter *et al*, 1993). The risk of prostate cancer increases with the number of first or second-degree affected relatives from about two-fold with a single first-degree relative to 8.8 with a first- and second-degree relative (Carter *et al*, 1993).

There is little information concerning psychosocial and behavioural factors having an impact on PSA screening in men with a familial risk and the existing literature provides contradictory results. Studies have reported that men with a family history are more likely to have a PSA test (McDavid *et al*, 2000; Jacobsen *et al*, 2004), others found no association between family history and screening uptake (Taylor *et al*, 1999; Miller *et al*, 2001).

Research focusing exclusively on men with a family history of prostate cancer has suggested that older age (men aged 50 and over), knowledge of recommended screening frequency, high levels of education, discussion with a physician, no co-morbidity, and having more affected relatives are associated with greater screening uptake (Bratt *et al*, 1997, 2000; Cormier *et al*, 2003).

A few studies have investigated psychological morbidity, screening behaviour and perceived risk in first-degree relatives (FDRs). In general, psychological morbidity is low in FDRs of prostate cancer patients. However, high trait anxiety, previous screening, having a son, perceived high risk, having more than one affected FDR, and being younger than the affected relative (index case) have all been associated with greater psychological morbidity and cancer specific worry (Bratt *et al*, 2000; Cormier *et al*, 2002; Beebe-Dimmer *et al*, 2004). In relation to screening behaviour, men with higher levels of cancer-specific stress are less likely to opt for screening (Bratt *et al*, 2000). Moreover, greater perceived efficacy regarding screening appears to be associated with subsequent PSA testing (Vadaparampil *et al*, 2004).

In addition to some of the contradictory findings in the literature, the absence of an association between perceived susceptibility and screening behaviour, runs counter to theoretical models of health behaviour (Rosenstock, 1974a, 1974b). When interpreting these contradictory findings, the methodological difficulties associated with previous studies need to be considered. Many studies have been limited by a focus on assessing intention to take a PSA test, instead of measures of actual screening behaviour. Further, some studies use a retrospective design relying on self-reports of previous screening as their sole measure of screening behaviour.

The present study aimed to address some of the limitations of earlier studies by examining psychosocial attributes, past screening behaviour, and adherence to a PSA familial screening feasibility study (FSFS) – a measure of ‘actual’ screening behaviour.

Five research questions are addressed:

1. What are the levels of psychological morbidity in a cohort of men with a family history of prostate cancer entering a familial PSA screening study?
2. How is perceived risk of developing prostate cancer related to psychological morbidity, past screening behaviour and adherence to a familial screening study?
3. What is the relationship between perceived barriers, benefits, and knowledge of PSA screening, perceived susceptibility to prostate cancer, and psychological morbidity, and is this related to past screening behaviour or adherence to a screening programme?
4. What reasons do men give for entering a familial PSA screening study and are these related to perceived risk, psychological morbidity, past screening behaviour, and screening adherence?
5. What effect do socio-demographic variables have on psychological morbidity, perceived barriers, benefits, and knowledge, and susceptibility, perceived risk, reasons for having test, past screening behaviour, and adherence to a familial screening programme?

## METHODS

### Participants

Participants were FDRs of young (cancer diagnosed at 65 years or less) prostate cancer patients diagnosed within the last 4 years. Patients were identified through cancer registries, British Association of Urological Surgeons (BAUS), the National Cancer Research Network (NCRN) and participating hospital departments. (see Melia *et al*, linked paper in this issue for more details of recruitment). Eligible FDRs were: (1) aged from 45 to 69 inclusive; (2) disease free; (3) residing in the UK; (4) able to complete their questionnaires in English.

### Procedure

The recruitment of FDRs into the present study followed on from their recruitment into a linked PSA FSFS (see Melia *et al*, 2006). For the purpose of the study, index cases (ICs) were contacted who had given details of their FDRs to the FSFS. ICs were sent separate information on the psychosocial study and asked for their consent to contact FDRs with regards to participation in the linked psychosocial study. After ICs gave consent, FDRs were contacted either by sending further information and a questionnaire or by a phone call from the researcher, according to the IC's preference. If interested in the study, FDRs who had been phoned were then sent an information pack and questionnaire. All questionnaires were completed before PSA screening.

The present study was approved by the South London Multi-centre Research Ethics Committee (MREC 00/1/73) and all ICs and FDRs gave written informed consent.

### Measures

These covered levels of psychological morbidity, barriers, benefits, and knowledge of PSA screening and perceived susceptibility to prostate cancer.

#### Psychological morbidity

Four previously validated measures were used: the General Health Questionnaire 12 (GHQ12), the Impact of Events Scale (IES), the Cancer Worry Scale-Revised (CWS-R) and Health Anxiety Questionnaire (HAQ).

The GHQ12 (Goldberg and Hillier, 1979) is an index of nonspecific psychological morbidity developed for use in community surveys. A cutoff score of 3 or more indicates psychiatric ‘caseness’; a threshold previously derived from general practice samples. The IES (Horwitz *et al*, 1979) assesses intrusive and or avoidant thoughts modified to relate to risk of prostate cancer and has been adapted for use with high-risk populations to reflect subjective stress due to familial cancer experiences; a high score indicates frequent intrusive/avoidant thoughts about risk of cancer. The CWS-R (Lerman and Schwartz, 1993) assesses degree of worry about developing cancer and includes two additional items: (Watson *et al*, 1999) assessing frequency of worry and the extent to which worry is a problem. A high score indicates greater worry but no clinical cutoff points are currently available.

Health anxiety and behaviour were assessed using two subscales from the HAQ (Lucock and Morley, 1996). This 21-item scale identifies individuals with high levels of concern about their health and includes four subscales. The two subscales included in the present study were: (1) Health Worry and Preoccupation and (2) Reassurance Seeking Behaviour. The other two subscales – Fear of Illness and Death and Interference with Life – duplicate other questions asked of the participants or were inappropriate for the study questions. The abbreviated HAQ uses 11 of the original 21 items with a high score indicating greater anxiety regarding an individual's health.

#### Barriers to PSA screening

This was assessed using the Perceived Barriers Scale (PBS) – a nine-item measure. (Kash *et al*, 1992; Watson *et al*, 1999), covering physical discomfort, fear of examination, transport to screening clinic, distress caused by screening, and taking time from work/family/social obligations to attend. A high score indicates greater perceived barriers to PSA screening.

#### PSA screening – benefits, knowledge and susceptibility to prostate cancer

Participants rated statements about managing risk of prostate cancer and perceived benefits to screening, perceived risk of prostate cancer, knowledge and a study specific question assessing participant's confidence in the PSA test results. The 10 items, derived from Kash *et al* (1992) and Watson *et al* (1999) were assessed using a 4-point scale ranging from ‘strongly agree’ to ‘strongly disagree’. A high score indicates: (1) greater perceived benefits regarding PSA testing, (2) greater knowledge regarding PSA screening and prostate cancer, and (3) greater perceived susceptibility to developing prostate cancer.

#### Prostate cancer relative risk perceptions

Relative perceived risk of developing prostate cancer was assessed using a single question: ‘What do you think your risk is compared with the average man of your age?’ This was rated on a 5-point scale ranging from ‘Very much lower than average’ to ‘Very much higher than average’.

#### Demographic variables

Socio-demographic variables included: age, ethnicity, marital status, education, employment status, number of children, and religion.

In addition, the assessment included details of past screening behaviour, present screening behaviour (as part of FSFS), family history of prostate cancer (e.g. number of relatives diagnosed with prostate cancer), reason for having a PSA test, impact of being invited into a PSA screening study, and family history of other types of cancer.

### Statistical methods

A univariate analysis (*P*⩽0.05) comparing men with self-reported past screening behaviour (digital rectal examination or ‘DRE’ or PSA) was carried out using *χ*^2^ tests of heterogeneity for categorical data and Independent *t*-tests (normally distributed) or Man–Whitney *U* tests (non-normally distributed) for continuous variables. Following the results of univariate analysis of psychological morbidity and screening adherence data, stepwise logistic regression modelling was carried out in order to identify the variables that best predicted psychiatric ‘caseness’ (score of 3 or more on the GHQ12), cancer worry (scores over the sample median), high subjective stress (total IES scores above the mean (8.6) reported in a previous study) (Bratt *et al*, 2003), and adherence to familial screening (present screening practice as part of the FSFS). Scores for continuous variables were quartiled and treated as categorical variables for the purpose of regression modelling. In addition, due to the low internal consistency of the perceived benefits, knowledge, and susceptibility subscales – as measured by a Cronbach's alpha coefficient of 0.16, 0.40, and 0.31 respectively – these items were entered separately into the regression models. As the measures of psychological morbidity were not normally distributed median GHQ, IES and CWS-R scores will be reported along with their associated interquartile range (IQR). Mean and standard deviation will be reported for the IES to enable comparison with previous findings. All analysis was carried out using a commercially available statistical software package – SPSS 12.01.

## RESULTS

Of 255 ICs (see FSFS reference for details), 155 (61%) consented to us contacting their male FDRs. This yielded a total of 207 FDRs who were approached to participate. In total, 62% (*n*=128) consented and filled in baseline measures, 2.5% (*n*=5) refused participation, and 36.5% (*n*=74) did not respond to their letter of invitation.

Table 1 shows the socio-demographic characteristics of the study sample. The mean age of the sample was 58 years. (s.d.=6). Participants were mainly of White ethnicity (95%), employed or retired (91%), and married or cohabiting (88%). Most were from social classes I & II (64%) and reported having children (80%) and 35% reported having at least one male child.

In total, 105 men (82%) reported having one brother with prostate cancer. Two men (1.5%) reported having only a father with prostate cancer. 19 (15%) reported having a brother and father with the disease. There were two men (1.5%) who were unaware as to who exactly had prostate cancer in their family, despite being in a familial screening study. In addition, 61 men (48%) reported having a family history of cancer, other than prostate.

In total, 80 men (63%) reported some previous screening for prostate cancer, 52 reported PSA, 21 reported a digital rectal examination (DRE) and seven did not specify type of screening. Previous screening was associated with having a brother and father with prostate cancer (*χ*^*2*^_exact_, *P*=0.003), higher social class (*χ*^*2*^_exact_, *P*=0.01), having some form of qualification (*χ*^*2*^_exact_, *P*=0.001), agreeing to take part in the familial screening programme to ‘get more information regarding prostate cancer’ (*χ*^*2*^_exact_, *P*=0.02), having a realistic or elevated sense of perceived risk (*χ*^*2*^_exact_, *P*=0.001) and higher perceived benefits of PSA screening (*t*-*test*, *P*=0.05). There were no significant differences (*P*=0.05) in psychological morbidity (GHQ, IES, CWS-R, HAQ) between those reporting past screening and those who did not report any previous screening behaviour.

### Psychological morbidity

A total of 18 (14%) men scored above the GHQ12 threshold for psychiatric ‘caseness’ (median=0, IQR 0–1). Internal consistency as measured by Cronbach's alpha coefficient was good (0.89).

Univariate analysis revealed that higher IES score (*P*=0.04), being unemployed (*P*=0.002), reporting being a little worried or anxious since invitation to screening (*P*=0.05), agreeing to take part in the familial screening programme ‘for the sake of your relative who has cancer’ (*P*=0.02), family history of cancer other than prostate (*P*=0.03), and higher cancer specific worry (*P*=0.008) were all associated with psychiatric ‘caseness’. The multivariate stepwise regression model revealed that being unemployed, family history of cancer (other than prostate) and higher cancer specific worry reliably predict psychiatric ‘caseness’. The other significant univariate variables were not retained in the final model (Table 2).

In total, 38 men (30%) scored above the median for subjective stress on the IES (median=2, IQR 0–11). The mean for total IES was 7.59, s.d.=11.67. Internal consistency for the total IES as measured by Cronbach's alpha coefficient was good (0.93). 0.87 and 0.88 for the intrusiveness and avoidance subscales respectively.

Univariate analysis revealed that higher cancer specific worry (*P*=0.001), psychiatric ‘caseness’ on the GHQ12 (*P*=0.04), finding the psychosocial evaluation itself helpful (*P*=0.02), reporting being a little worried or anxious since invitation to screening (*P*=0.001), higher perceived susceptibility (*P*=0.03), greater perceived barriers (*P*=0.02), higher health anxiety (*P*=0.001), were associated with higher IES scores. Multivariate stepwise regression modelling revealed that higher cancer specific worry, (*P*=0.001) finding the psychosocial evaluation itself helpful, (*P*=0.02) reporting being a little worried or anxious since invitation to screening (*P*=0.01) and higher perceived susceptibility (*P*=0.005) reliably predict high IES scores. The other significant univariate variables were not retained in the final model (Table 3).

The revised six-item Cancer Worry Scale (CWS-R) yielded good internal reliability=0.82. The median for the CWS-R was 8 and the IQR was 7–10.

Higher health anxiety (*P*=0.001), perceived barriers (*P*=0.001), higher perceived susceptibility (*P*=0.02), higher perceived knowledge (*P*=0.007), higher IES (*P*=0.001), psychiatric ‘caseness’ (*P*=0.03), having a brother and father with prostate cancer (*P*=0.02), realistic perceived risk compared to an underestimation of personal risk (*P*=0.05), and agreeing to take part in the familial screening programme ‘for your own sake’ (*P*=0.04) were all associated with higher cancer-specific worry. The multivariate stepwise regression model revealed that higher health anxiety, moderate *vs* low perceived barriers to PSA screening, higher perceived susceptibility, higher subjective stress scores (IES) and realistic perceived risk compared to underestimation of risk all reliably predict higher cancer specific worry regarding prostate cancer. The other significant univariate variables were not retained in the final model (Table 4).

### Adherence to FSFS

In total, 10 men (8%) failed to adhere to the FSFS. The relationship between family history, psychosocial and behavioural variables and adherence to familial screening was examined using *χ*^*2*^ and logistic regression analysis. A multivariate model was not constructed as only one variable – past screening behaviour – reliably predicted (*P*=0.04) men's adherence to familial screening, that is, men who reported having any kind of screening (DRE or PSA) for prostate cancer were more likely to adhere to the FSFS (Table 5).

## DISCUSSION

Our goals for this study were to examine the factors relating to psychological morbidity and screening adherence in a group of FDRs entering a PSA FSFS.

The findings indicate that FDRs entering the FSFS have quite low levels of psychological morbidity; 18 men (14%) scored above the GHQ12 threshold for psychiatric ‘caseness’ and this is within the normal population range (Jenkins *et al*, 1997). Psychiatric ‘caseness’ was predicted by family history of cancer other than prostate, higher cancer specific worry, and being unemployed. Further, there were moderately high levels of subjective stress regarding prostate cancer. In total, 30% of men scored high on subjective stress as measured by the IES (defined as above the mean IES score (8.6) reported by Bratt *et al*, 2003). Higher cancer specific worry, higher perceived susceptibility, and reporting being a little worried or anxious since invitation to screening were found to reliably predict high subjective stress regarding prostate cancer. Interestingly, men reporting that they found the psychosocial evaluation itself helpful also reliably predicted high subjective stress as measured by the IES. It is possible that men who have high levels of subjective stress seem to appreciate an evaluation of their psychological well being as they enter a screening programme, although this requires further investigation.

Levels of cancer-specific worry are lower in this cohort than those previously reported in other familial cancer studies: median=8 compared to scores for a cohort offered BRCA1/2 predictive genetic testing, median=11 (Foster *et al*, 2002). This may be due to gender differences in both reporting both the experience and reporting of worry (Moynihan, 1998). Cancer-specific worry was reliably predicted by realistic perceived risk compared to an underestimation of risk, higher perceived barriers to screening, higher health anxiety, higher subjective stress and higher perceived susceptibility.

In relation to screening adherence to an FSFS, the present study found that only previous screening behaviour reliably predicts adherence to the familial screening study. First-degree relatives who reported no previous screening were significantly more likely to drop out of the screening programme, that is, fail to have their actual PSA test having consented to participate.

In relation to screening behaviour, the present study found a high (63%) reported level of previous screening in FDRs entering the FSFS. This was much higher than anticipated and thus motivated an *aposteriori* analysis of the association between previous screening and other psychosocial and socio-demographic variables. Reported previous screening behaviour was found to be associated with having more than one FDR with prostate cancer, having a realistic or elevated sense of perceived risk, higher perceived benefits of PSA screening, higher perceived susceptibility to prostate cancer, higher social class and having some form of qualification.

Our findings regarding psychological morbidity concur with previous research showing that levels of psychological morbidity in FDRs of prostate cancer patients are not unusually high (Bratt *et al*, 2000; Cormier *et al*, 2002), although a minority (30%) report some symptoms of stress as assessed by the Impact of Evens Scale, which assessed specific intrusive thoughts or avoidant reactions to concern about risk of prostate cancer. Therefore, it is less that these FDRs have higher than average levels of psychiatric caseness and more that they have some level of stress, in a minority, specific to prostate cancer risk. How such concerns might be addressed requires some consideration. In addition, the current study reinforces previous findings regarding the importance of health anxiety, perceived risk, and having more than one affected FDR in relation to psychological morbidity (Bratt *et al*, 2000; Cormier *et al*, 2002; Beebe-Dimmer *et al*, 2004).

Our finding regarding the importance of employment status in relation to psychological morbidity is in keeping with previous findings suggesting the importance of work to both men's mental health and health behaviours (Lahelma, 1992; Artazcoz *et al*, 2004). The present study found that increased reporting of perceived barriers to PSA screening such as taking time off work is associated with higher levels of cancer-specific worry. Some investigation of the specific methods that might allow a reduction in barriers to screening in those men who may otherwise see a benefit to screening is indicated.

Cormier *et al* (2002) found a significant association between having a son and psychological morbidity; however, we found no such relationship. This inconsistency may be explained by Cormier *et al*'s focus on worry about genetic susceptibility and genetic testing. Those measures explicitly assessed the amount of worry over genetic heritability with regards to participants’ children. This is distinctly different from the measures of psychological morbidity in the present study, which focused on the participants’ personal cancer worry.

With respect to screening behaviour, Vadaparampil *et al* (2004) found that previous screening predicted participation in subsequent PSA screening. However, unlike the present study, the authors found participation to be related to older age (⩾50), annual income (⩾US$40 000) and higher levels of self-efficacy. Both studies show that previous screening behaviour predicts present screening behaviour. However, the present study did not measure levels of income or self-efficacy, although we did collect data on social class/occupation, and this was not found to be associated with screening adherence. Inconsistencies may also be due to methodological differences including the time frames and context in which the screening took place. Our study reports screening as part of the FSFS (adherence) while Vadaparampil *et al* (2004) conducted a retrospective study in which they contacted FDRs 14 months after their initial assessment to find out if they had undertaken PSA screening. Only one previous study has investigated adherence to a familial PSA screening programme (Roumier *et al*, 2004). The authors found that younger men, those who were more anxious and men with more than one affected FDR were less likely to adhere to a familial screening program. The present study failed to confirm such associations. However, Roumier and co-workers examined the adherence of FDRs to a second and third annual PSA screening test, after an initial test, in contrast to our study, which examined adherence to a one off PSA test after agreeing to take part in a FSFS.

Our *aposteriori* analyses regarding the association between previous screening and other psychosocial and socio-demographic variables are consistent with findings reported elsewhere (Bratt *et al*, 1997, 2000; Cormier *et al*, 2003; Vadaparampil *et al*, 2004). However, unlike previous studies (Bratt *et al*, 2000; Miller *et al*, 2001; Vadaparampil *et al*, 2004), we found no association between age and past screening behaviour of FDRs. This inconsistency may be due to differences in the status and awareness of PSA screening and variations in endorsement by health care providers, both of which can be seen to vary between the UK and other countries such as the US where for the latter, the culture of PSA screening is more widespread (O’Dell *et al*, 1999).

The present study found that agreeing to take part in the familial screening programme to ‘get more information regarding prostate cancer’ was also associated with previous screening behaviour. This suggests that there are cohorts of men who are actively engaged in help-seeking behaviours. For example, men may want to find out about new developments. Alternatively, men may not have been given sufficient information when they were screened previously. It may be useful for health care providers to highlight information provision as a priority when attempting to implement a screening programme, whether a man has had previous screening or not.

The present study offers some insights into FDRs’ screening behaviour, psychological morbidity and adherence to a familial screening programme, but some study limitations must also be taken into consideration. Due to the small number of people that did not adhere to screening (*n*=10), it is likely that our model to predict adherence is underpowered, although the findings are consistent with previous research (Roumier *et al*, 2004).

Our response rates from ICs and FDRs were 61 and 62%, respectively. The men recruited to the present study were only a percentage (75%) of those men who had consented to the FSFS. Our data may not extrapolate more broadly as the sample consisted of predominantly Caucasian men in upper social class groups. Black (African and African-Caribbean ethnicity) men did not form part of the sample for either the present study or the FSFS despite the evidence that these men may be at an additional increased risk of prostate cancer (Grulich *et al*, 1992; Chinegwundoh *et al*, 2003). As suggested by other research, ‘traditional’ methods of recruitment may need to be changed if men from these groups are to be recruited into similar studies (Odedina *et al*, 2002, 2004). Understanding why there is a relatively low response rate of men in general into research screening is needed. It is conceivable that nonparticipants are anxious or not receptive to research or particular methods of screening such as DRE (Odedina *et al*, 2004). Aspects of masculinity such as appearing weak rather than strong and healthy, may be impacting on participation in screening as shown elsewhere in relation to health practices (Connell, 1995; Moynihan, 1998; Courtenay, 2000), and some of these men may require targeted counselling. The uncertainty surrounding treatment and screening for prostate cancer may have acted as a possible barrier to screening in some men (Chapple *et al*, 2002).

In conclusion, although levels of psychological morbidity appear to be quite low in the men attending the prostate familial screening programme, certain factors such as health anxiety, cancer worry, unemployment and perceived risk do impact on their state of well being. Previous screening behaviour predicts current and likely future uptake of screening. Thus, eliciting men into screening (where this may be beneficial) in the first place remains a challenge. Further information on PSAS screening costs–benefits is likely to be helpful both in facilitating decision-making and assuaging specific cancer worry. More research is needed on decision making in PSA screening uptake before it is possible to provide specific recommendations on service development. Although reasons cannot be provided, the finding that men without a previous history of testing are more likely to be those who do not adhere to familial screening, despite consenting, suggests that this group may also require additional information.

## Figures and Tables

**Table 1:** Socio-demographic data for 128 unaffected

**Variable**	***N***	**(%)**
*Age (years)*		
45–49	11	9
50–59	62	48
60–69	55	43
		
*Ethnicity*		
White	122	96
Asian	3	2
Chinese/other	3	2
		
*Social class*		
I	16	13
II	65	51
III	31	24
IV	11	9
V	4	3
		
*Marital status*		
Married/cohabiting	113	88
Single/divorced/widowed	15	12
		
*Employment status*		
Employed	74	58
Retired	43	33
Unemployed	11	9
		
*Education*		
Higher	28	22
Further	24	19
Secondary	39	30
None	37	29
		
*Any children*		
Yes	103	80
No	25	20
		
*Male children*		
Yes	45	35
No	83	65

**Table 2:** Stepwise logistic regression model of significant predictors of psychiatric ‘caseness’

**Variable**	**Odds ratio**	**(95% confidence intervals)**	***P*** **-value**
Family history of cancer other than prostate	6.15	(1.43–26.37)	0.02
Higher cancer specific worry	13.12	(1.96–87.97)	0.008
Being unemployed	16.53	(2.70–101.38)	0.002

**Table 3:** Stepwise logistic regression model of significant predictors of high subjective stress regarding prostate cancer

**Variable**	**Odds ratio**	**(95% confidence intervals)**	***P*-value**
Higher cancer specific worry	18.31	(3.70–90.56)	0.001
Higher perceived susceptibility	2.82	(1.37–5.78)	0.005
Finding psychosocial evaluation helpful	4.46	(1.27–15.64)	0.02
A little worried or anxious since invitation	3.74	(1.32–10.59)	0.01

**Table 4:** Stepwise logistic regression model of significant predictors of high cancer specific worry regarding prostate cancer

**Variable**	**Odds ratio**	**(95% confidence intervals)**	***P*** **-value**
Higher subjective stress	10.63	(2.91–38.90)	0.001
Higher perceived susceptibility	12.71	(1.14–141.39)	0.04
Realistic perceived risk *vs* underestimate	4.08	(1.31–12.66)	0.02
Moderate *vs* low perceived barriers	7.04	(1.62–30.61)	0.009
Higher health anxiety	14.99	(3.27–68.64)	0.001

**Table 5:** Logistic regression model of significant predictor of screening adherence

**Variable**	**Odd ratio**	**(95% confidence intervals)**	***P*** **-value**
Previous screening behaviour	4.38	(1.08–17.85)	0.04
